# The deubiquitinase *USP54* is overexpressed in colorectal cancer stem cells and promotes intestinal tumorigenesis

**DOI:** 10.18632/oncotarget.12769

**Published:** 2016-10-19

**Authors:** Julia M. Fraile, Diana Campos-Iglesias, Francisco Rodríguez, Yaiza Español, José M.P. Freije

**Affiliations:** ^1^ Departamento de Bioquímica y Biología Molecular, Facultad de Medicina, Instituto Universitario de Oncología, Universidad de Oviedo, Oviedo, Spain

**Keywords:** degradome, CSCs, ubiquitin-specific proteases, gene targeting

## Abstract

Ubiquitin-Specific Proteases (USPs) are deubiquitinating enzymes frequently deregulated in human malignancies. Here, we show that *USP54* is overexpressed in intestinal stem cells and demonstrate that its downregulation in colorectal carcinoma cells impedes tumorigenesis. We have generated mutant mice deficient for this deubiquitinase, which are viable and fertile, and protected against chemically-induced colorectal carcinoma. Furthermore, we show that *USP54* is upregulated in human colon cancer and associates with poor prognosis. In agreement with these results, *Usp54* downregulation in mouse melanoma cells inhibits lung metastasis formation. Collectively, this work has uncovered the pro-tumorigenic properties of USP54, highlighting the importance of deubiquitinating enzymes as promising targets for the development of specific anti-cancer therapies.

## INTRODUCTION

Proteases have essential functions in the regulation and execution of most physiological processes and pathological conditions [1–3]. Consequently, alterations in their structure or regulation are associated with diverse pathologies, such as arthritis, neurodegenerative alterations, cardiovascular diseases and cancer. In this sense, proteases have raised remarkable interest as potential targets of anticancer therapies [4–6]. Nevertheless, the success of these strategies has been limited by the complexity of the entire protease repertoire, defined as degradome, and by the fact that not all proteases exhibit pro-tumorigenic roles in human malignancies, as the list of human proteases with tumor suppressive roles in cancer has been growing over the last decades [7].

Among all proteases whose function is involved in cancer regulation, deubiquitinases or DUBs are of particular interest due to their wide functional diversity [8, 9]. DUBs are critical regulators of ubiquitin-mediated signaling pathways because of their ability to cleave the isopeptide bond that links ubiquitin chains to target proteins. Based on structural and sequence similarities, DUBs are grouped into six families: ubiquitin-specific proteases (USPs), ubiquitin carboxy-terminal hydrolases (UCHs), ovarian-tumor proteases (OTUs), Machado-Joseph disease protein domain proteases (MJDs) and monocyte chemotactic protein-induced proteins (MCPIPs), all of them cysteine proteases, and JAMM/MPN domain-associated metallopeptidases, which is the only family that belongs to the catalytic class of metalloproteases [10]. With more than 50 members, USPs constitute the largest family of DUBs [11], and have a profound impact on the regulation of multiple biological processes that are frequently altered in cancer [10]. Previous studies have reported that USPs exhibit tumor suppressing or oncogenic functions due to mutations or changes in their expression levels. Furthermore, there are USPs with pro-tumorigenic and anti-tumorigenic activities depending on the cellular context or the target affected by their regulation [12, 13]. In this regard, sequence alterations of USP54 have been recently described in human acute lymphoblastic leukemia [14]. However, the functional relevance of this deubiquitinating enzyme in solid tumors has not been explored so far.

In this work, we have demonstrated that *USP54* is overexpressed in intestinal cancer stem cells (CSCs) and that its downregulation reduces the tumorigenicity of colon cancer cells both *in vitro* and *in vivo*. To further investigate the *in vivo* function of USP54 in cancer, we have generated mutant mice deficient for this DUB. These mice are viable and fertile with no obvious abnormalities. We have found that USP54 deficiency protects against azoxymethane-induced colon carcinoma and inhibits metastasis formation by melanoma cells, indicating that this DUB functions as an oncogenic factor in both malignancies. Finally, we show that high levels of USP54 are a poor prognosis marker in colorectal carcinoma, suggesting the interest of this enzyme as a potential target of anticancer therapies.

## RESULTS

### *USP54* is overexpressed in intestinal stem cells and promotes cancer progression

**Figure 1:**
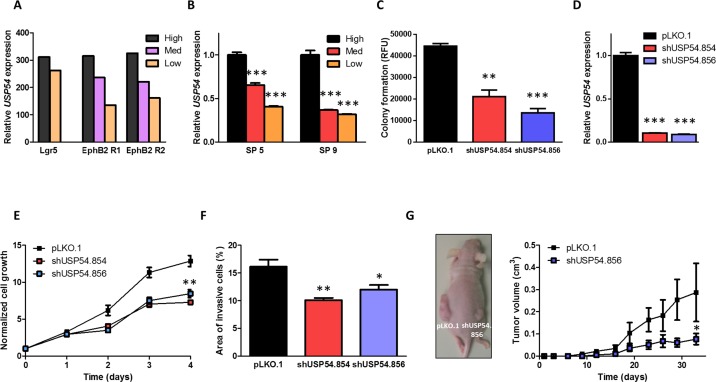
*USP54* is upregulated in intestinal stem cells and induces colorectal carcinoma progression **A.** Analysis of *USP54* expression from microarray data (GEO accession number GSE27605). High, high Lgr5 or EphB2 receptor expression levels; Med, medium EphB2 receptor expression levels; Low, low Lgr5 or EphB2 receptor expression levels. **B.** qRT-PCR analysis of *USP54* expression in two different colorectal carcinoma xenografts (SP5 and SP9), two-tailed Student's t-test (***, *P* < 0.001). **C.** Anchorage-independent growth of control (pLKO.1) and USP54-depleted HCT116 cells (shUSP54.854 and shUSP54.856), two-tailed Student's t-test (**, *P* < 0.01; ***, *P* < 0.001). RFU, relative fluorescence units. **D.** qRT-PCR analysis of *USP54* expression in HCT116 cells transduced with control (pLKO.1) or *USP54*-specific shRNAs (shUSP54.854 and shUSP54.856). Statistical significance was assessed by two-tailed Student's t-test (***, *P* < 0.001). **E.** MTT proliferation analysis of wild-type and USP54-deficient HCT116 cells, Mann Whitney-Wilcoxon test (**, *P* < 0.01). **F.** Average area of invasion of HCT116 cells transduced with empty vector (pLKO.1) or *USP54*-specific shRNAs (shUSP54.854 and shUSP54.856). Mann Whitney-Wilcoxon test was used to analyze statistical significance (*, *P* < 0.05; **, *P* < 0.01). **G.** Tumor xenograft model performed with subcutaneously injected control and USP54-depleted HCT116 cells. Data are presented as mean ± SEM and statistical significance was assessed by using a non-parametric Mann Whitney-Wilcoxon test (*, *P* < 0.05).

To explore the role of DUBs in cancer stem cell biology, we have analyzed the expression of several DUBs in publicly available transcriptional profiles of intestinal epithelial cells, which had been FACS-sorted according to high, medium or low EphB2 receptor levels to obtain intestinal stem cell (ISC)-enriched cell populations [15]. This analysis revealed that *USP54* was overexpressed in cells with high expression of both Lgr5 and EphB2 receptor, supporting that *USP54* expression is upregulated in intestinal cells with stem properties (Figure 1A). We then validated these results in cells derived from primary tumors expanded as subcutaneous xenografts in immunodeficient mice and sorted in three populations displaying high, medium or low EphB2 receptor expression. Similarly, *USP54* was upregulated in the samples with high EphB2 receptor levels (Figure 1B), suggesting a role of USP54 in maintaining the stem characteristics of both ISCs and CSCs. In agreement with these results, silencing of *USP54* in HCT116 colorectal carcinoma cells with two different shRNAs (shUSP54.854 and shUSP54.856), reduced their ability to grow in soft agar (Figure 1C). Downregulation of *USP54* expression was verified by qRT-PCR (Figure 1D). Moreover, the proliferation rate of HCT116 cells was decreased upon USP54 depletion, compared to wild-type cells (Figure 1E). Similarly, *USP54* downregulation decreased the invasiveness of HCT116 cells (Figure 1F). Consistent with these findings, depletion of USP54 decreased the tumorigenicity of HCT116 when injected into nude mice (Figure 1G). Altogether, these data support the role of USP54 in promoting colorectal carcinoma and suggest a new function of USP54 in controlling the stem properties of intestinal cells.

### Usp54 is dispensable for embryonic development and normal growth of adult mice

**Figure 2:**
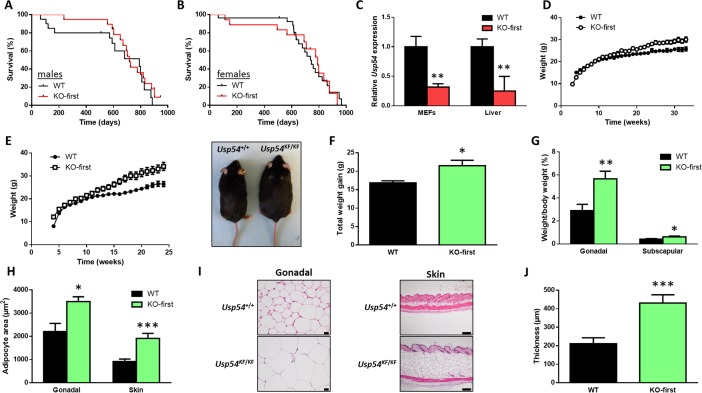
Usp54 is dispensable for embryonic development and adult mice lifespan **A.**, **B.** Kaplan-Meier survival curves for wild-type and Usp54-deficient males (A) and females (B). **C.** TaqMan-based qRT-PCR analysis of *Usp54* in MEFs and liver tissues from *Usp54*^+/+^ and *Usp54*^KF/KF^ mice. Data are represented as relative quantification, RQ ± SEM, two-tailed Student's t-test (**, *P* < 0.01). **D.** Body weight curves of *Usp54*^+/+^ and *Usp54*^KF/KF^ female mice kept on standard diet. **E.** Body weight curves of *Usp54*^+/+^ and *Usp54*^KF/KF^ female mice kept on high-fat diet and a representative image of females of each genotype at the end of the experiment. **F.** Total weight gain in the same animals. **G.** Percentage of gonadal and subscapular fat mass with respect to total body weight of the same animals. **H.** Mean adipocyte area in gonadal and skin fat. **I.** Representative histological images. Scale bar: 20 μm (gonadal fat) and 200 μm (skin fat). **J.** Average thickness of the subcutaneous fat deposits for each genotype. Statistical significance was assessed by a non-parametric Mann Whitney-Wilcoxon test (*, *P* < 0.05; **, *P* < 0.01; ***, *P* < 0.001).

To further evaluate the role of USP54 in cancer, we generated a mouse model deficient for this DUB by using a knockout-first (KF) strategy. Mice homozygous for the KF allele, hereafter referred as *Usp54*^KF/KF^, were viable and fertile without any obvious alteration. Thus, we did not find any difference in survival between *Usp54*^+/+^ and *Usp54*^KF/KF^ mice, neither in males (Figure 2A) nor females (Figure 2B). Using qRT-PCR in mouse embryonic fibroblasts (MEFs) and liver tissue samples, we have demonstrated that *Usp54*^KF/KF^ mice showed a reduced, but not abolished, expression of *Usp54*, compared to wild-type animals (Figure 2C). These results suggested that *Usp54*^KF/KF^ mice presented a hypomorphic phenotype. We next crossed these animals with mice expressing Cre recombinase to generate the KO mice (*Usp54*^Δ/Δ^) through the elimination of *Usp54* exons 4 to 6. These animals were viable and fertile with no obvious abnormalities, demonstrating that Usp54 is dispensable for embryonic and adult mice development. In the course of phenotypic characterization of *Usp54*^KF/KF^ mice, we found a slight increase in body weight in female mice as compared with their wild-type littermates kept on standard chow (Figure 2D). Interestingly, differences in body weight augmented when both Usp54-deficient and wild-type female animals were fed a high fat diet (Figure 2E and 2F). Accordingly, *Usp54*^KF/KF^ female mice kept on high-fat diet showed a significantly larger increase in gonadal and subscapular weight fat pads, compared to wild-type animals (Figure 2G). Thus, histological analysis of white adipose tissue from both gonadal and subcutaneous fat deposits revealed that *Usp54^KF/KF^* mice showed higher adipocyte area than *Usp54^+/+^* mice (Figure 2H and 2I). In agreement with these data, subcutaneous fat deposits were significantly thicker in *Usp54^KF/KF^* female mice (Figure 2I and 2J). Collectively, these results demonstrate that Usp54-deficiency is compatible with normal mouse development and life, although in female animals it leads to an increase in body weight due to fat accumulation.

### USP54 promotes cancer invasion in chemically-induced colorectal carcinomas

**Figure 3:**
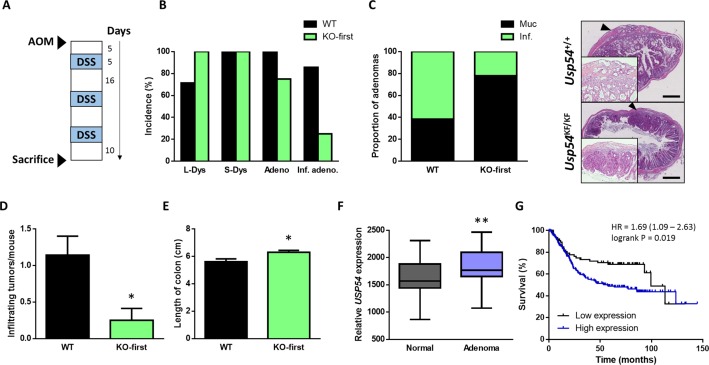
USP54 deficiency prevents azoxymethane-induced infiltrating colon tumors **A.** Schematic representation of azoxymethane-induced colon tumor protocol. **B.** Percentage of animals with the indicated histological alterations. L-Dys, light dysplasia; S-Dys, severe dysplasia; Adeno, adenocarcinomas; Inf. Adeno., infiltrating adenocarcinomas. **C.** Percentage of infiltrating and mucosal tumors within all carcinomas of each genotype and representative histological images. Scale bar: 500 μm. **D.** Average number of adenocarcinomas per mouse. **E.** Length of the colon at the end of the experiment. **F.** Analysis of *USP54* expression from 32 colorectal cancer patient samples, comprising pairs of tumor and matched normal mucosa (GEO accession GDS2947). **G.** Kaplan-Meier survival plot for 269 patients with intestinal cancer grouped as a function of quantile expressions of *USP54*. Statistical significance was assessed by a non-parametric Mann Whitney-Wilcoxon test (*, *P* < 0.05; **, *P* < 0.01).

To investigate USP54 role in colorectal cancer development *in vivo*, we induced colon carcinomas in *Usp54*^+/+^ and *Usp54^KF/KF^* mice, using azoxymethane (AOM) and dextran sulfate sodium (DSS) (Figure 3A). All wild-type animals, but only 75% of Usp54-deficient mice, developed adenocarcinomas (Figure 3B). Considering all colorectal carcinomas, the proportion of infiltrating adenocarcinomas was lower in *Usp54^KF/KF^* mice, compared to wild-type animals (Figure 3C). Moreover, the number of infiltrating tumors per mouse was significantly decreased in Usp54-deficient animals, compared to control mice (Figure 3D). Additionally, we measured shortening of colon as a marker of inflammation and found that colon samples from wild-type mice where shorter than those from Usp54-deficient animals (Figure 3E), revealing a less severe colitis in the absence of this deubiquitinase. These data indicate that USP54 deficiency impedes the development of colorectal carcinoma.

To further clarify the relevance of USP54 in colon cancer, we analyzed the expression of this DUB in transcriptional data from colorectal adenomas and normal mucosa [16] (GEO accession GDS2947). We found a significant increase in *USP54* expression in adenomas compared to matched normal mucosa (Figure 3F). Moreover, analysis of reported data on survival of patients with intestinal cancer showed a positive correlation between high *USP54* expression and lower survival (Figure 3G). Collectively, these data, together with the above finding that *USP54* is overexpressed in ISCs, support a pro-tumorigenic effect of USP54 in colorectal carcinoma.

### USP54 is necessary for metastasis of melanoma cells

**Figure 4:**
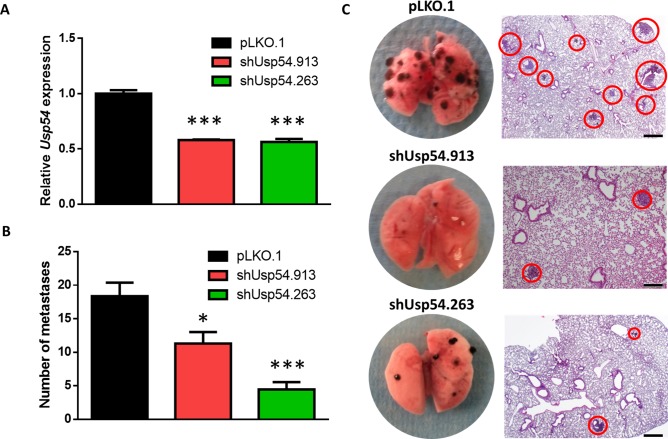
USP54 deficiency inhibits lung metastasis formation **A.** TaqMan-based qRT-PCR analysis of *Usp54* expression in B16F10 cells transduced with the indicated Usp54-specific shRNA or the empty lentiviral vector (pLKO.1) as a control. Data are represented as relative quantification, RQ ± SEM, two-tailed Student's t-test (***, *P* < 0.001). **B.** Number of metastases bigger than 200 μm in diameter. Statistical significance was assessed using a non-parametric Mann Whitney-Wilcoxon test (*, *P* < 0.05; ***, *P* < 0.001). **C.** Representative images of lungs and histological analysis for each condition. Scale bar: 200 μm.

**Figure 5:**
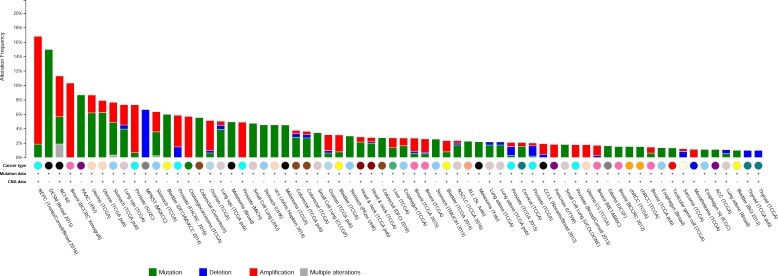
*USP54* alterations in human cancer Summary of *USP54* genetic alterations found in human cancer. ACC, Adrenocortical Carcinoma; Adeno, Adenocarcinoma; CCLE, Cancer Cell Line Encyclopedia; chRCC, Kidney Chromophobe; CS, Carcinosarcoma; DESM, Desmoplastic Melanoma; MPNST, Malignant Peripheral Nerve Sheath Tumor; NEPC, Neuroendocrine Prostate Cancer; PAAC, Acinar Cell Carcinoma of the Pancreas; SC, Small Cell; Sq and Squ, Squamous Cell Carcinoma; ucs, Uterine Carcinosarcoma. Data were obtained from cBioportal (http://cbioportal.org).

To further evaluate the role of USP54 in cancer progression, we analyzed *in vivo* the effect of *Usp54* knockdown on the formation of experimental lung metastasis by B16F10 murine melanoma cells. For this purpose, we transduced B16F10 cells with two different *Usp54*-specific shRNAs (shUSP54.913 and shUSP54.263) or the empty vector (pLKO.1) and we verified the downregulation of *Usp54* expression by qRT-PCR (Figure 4A). Then, we injected 25,000 cells through the jugular vein of 8-week-old C57BL/6N mice. After 21 days, the animals were sacrificed and their lungs were collected for histological analysis. Downregulation of *Usp54* decreased the number of metastases with more than 200 μm of diameter compared to pLKO.1-transduced control cells (Figure 4B and 4C). Interestingly, the inspection of publicly available cancer genome databases revealed frequent mutations in USP54, especially in melanoma and in pancreatic and endometrial carcinomas (http://cbioportal.org). It is also noteworthy the amplification of this gene in neuroendocrine prostate cancer and its deletion in malignant peripheral nerve sheath tumor (Figure 5). Collectively, these results support the essentiality of USP54 for lung metastasis formation *in vivo*, corroborating its oncogenic function in metastatic melanoma progression.

## DISCUSSION

The cancer stem cell (CSC) hypothesis proposes that tumors arise and are driven by a subset of cancer cells that retain stem cell properties [17, 18]. Over the last decades, several studies have addressed the role of DUBs in stem cell maintenance [19, 20]. In this context, we have analyzed the expression profile of several USPs in colorectal cancer stem cells, and we have found that *USP54* is consistently upregulated. Accordingly, we have shown that downregulation of *USP54* significantly decreases the tumorigenicity of colon cancer cells.

In this work, we have also generated an *Usp54*-deficient mouse model and we have shown that these animals are viable and fertile, demonstrating that Usp54 is dispensable for normal mouse development and survival. Interestingly, we have described the presence of metabolic alterations in *Usp54^KF/KF^* female mice characterized by an increase in body weight due to fat accumulation. By performing carcinogenesis protocols, we have demonstrated the oncogenic relevance of USP54 in colon cancer by finding that Usp54 deficiency protects against chemically-induced colorectal carcinoma. In agreement with these results, *USP54* is overexpressed in human colon cancer and those cases with higher expression levels of this gene present a poorer prognosis. These results, together with the finding that *USP54* is upregulated in colon CSCs, support the oncogenic function of this deubiquitinase in colorectal cancer progression. Additionally, we have found that *Usp54* downregulation impairs experimental lung metastasis formation by melanoma cells, corroborating the pro-tumoral effect of USP54 also in this pathology. In agreement with these results, mutations in *USP5*4 have been recently described in human leukemia, confirming the importance of this deubiquitinase in both hematological malignancies and solid tumors [14].

In summary, we have demonstrated that *USP54* is overexpressed in colon CSCs and promotes both colon carcinoma and melanoma progression. In this regard, future studies will be required to clarify the molecular mechanisms underlying the pro-tumoral effect of USP54 in cancer progression demonstrated in this work and to explore the translatability of these findings into clinical benefits for cancer patients.

## MATERIALS AND METHODS

### Generation of *Usp54* mice and genotyping

*Usp54*-mutant embryonic stem cells (ESCs) from the C57BL/6N mouse strain were purchased from KOMP. Two different clones of targeted ESCs (EPD0833_2_B10 and EPD0833_2_D09) were microinjected into C57BL/6N mouse blastocysts to produce chimeric mice that were then subsequently crossed with wild-type C57BL/6N mice to generate *Usp54*-heterozygous mice. The *Usp54*-KF allele was identified by PCR on genomic DNA from tail samples under the following conditions: denaturation at 94°C for 30 s, annealing at 60°C for 30 s, and extension at 72°C for 30 s, 30 cycles. We used the following primers for genotyping: wild-type-specific forward 5'-ccataatcccagcacctaagag-3', mutation-specific forward 5'-caagtgtggagggtggtgt-3', and common reverse 5'-ctggaagtaaacagaccggagt-3'.

### Animal care

All animal procedures were approved by the Committee for Animal Experimentation of the Universidad de Oviedo and performed in accordance with the guidelines of the Committee. Cre transgenic mice were obtained from Jackson Laboratory (No 006054). For diet-induced obesity, 4-week-old Usp54-deficient mice and their wild-type littermates were fed a high-fat diet containing 60% fat for 24 weeks (Research Diet, D12492). Mice were weighted once a week for all the duration of the experiment. For blood glucose measurements, blood from the tail vein was analyzed by using the Accu-Chek glucometer (Roche Diagnostics).

### Colon carcinogenesis protocol

For colon carcinogenesis induction, 9-week-old female mice were injected intraperitoneally with 12.5 mg/kg AOM (azoxymethane; Sigma-Aldrich, St. Louis, MO, USA). After 5 days, DSS (dextran sulfate sodium; MP Biomedicals) at 1.5% was administered in the drinking water for 5 consecutive days. Next, mice received reverse osmosis water for 16 days and two more DSS cycles at 1.5% were administered, with another interval of 16 days on water between them. 10 days after the last DSS cycle, mice were euthanized and colons were extracted, flushed with phosphate-buffered saline (PBS) and measured in length. The colons were fixed in 4% paraformaldehyde (PFA) and transversal sections were hematoxylin and eosin (H&E) stained. The tumors were counted and their histological features, as well as the grade of the inflammatory lesions, were analyzed for each mouse.

### Cell culture

Cancer cell lines HEK 293T and HCT116 were purchased from the American Type Culture Collection. Cells were routinely maintained in Dulbecco's modified Eagle's medium containing 10% fetal bovine serum, 100 U/ml penicillin, and 100 μg/ml streptomycin (Life Technologies).

### shRNA lentiviral infection

The two best shRNAs of both human and murine sets of 5 *USP54*-specific shRNA vectors, and empty vector, pLKO.1 (Open Biosystems, Thermo Scientific) were packaged in HEK 293T cells using a VSVG-based package system. After 24 h, viral supernatant was collected and added in a 1:3 dilution to previously seeded HCT116 or B16F10-luc2 cells, supplemented with 5 mg/ml of polybrene (Millipore). Stably transduced cells were selected with puromycin at a final concentration of 1 μg/ml.

### Real-time quantitative PCR (qRT-PCR) analysis

2 μg of total RNA from cells, isolated with RNeasy kit (Qiagen), was used to synthesize cDNA using the ThermoScript™ Reverse Transcriptase kit (Life Technologies). Then, qRT-PCR was performed using TaqMan^®^ gene expression assay for murine samples (*Usp54*, Mm00513373_m1) or Power SYBR^®^ Green PCR Master Mix for human cells (Life Technologies), using an Applied Biosystems 7300HT Real-Time PCR System. Relative expression was calculated as RQ=2^−ΔΔCt^.

### Soft agar colony formation

To determine anchorage-independent growth of HCT116 cells, 1,000 cells were seeded per well into 96-well plates and maintained during 8 days at 37°C and 5% CO_2._ Then, a CytoSelect™ 96-Well *In Vitro* Tumor Sensitivity Assay kit (Cell Biolabs) was used, following the manufacturer's instructions.

### Cell-proliferation assay

To quantify cell proliferation, we seeded 5,000 HCT116 cells transduced with either control (pLKO.1) or *USP54*-specific shRNAs per well (*n* = 6) into 96-well plates. Next, a Cell Titer 96 Non-Radioactive Cell Proliferation kit (Promega Corp.) was used following the manufacturer's instructions.

### Mouse xenograft model

Xenograft experiments were performed as previously described [21]. Briefly, 2 million control or *USP54*-silenced HCT116 cells were injected subcutaneously in both flanks of eight-week-old athymic Nude-Foxn1nu/nu mice (Charles River). Tumor size was measured twice per week using a caliber and mice were sacrificed 32 days post-injection.

### Lung metastasis protocol

For lung metastasis experimental model, 25,000 control or *Usp54*-silenced B16F10-luc2 tumor cells were injected through the jugular vein of previously anesthetized mice. After 3 weeks, mice were sacrificed and lungs were collected, fixed in 4% PFA in PBS and stored in 70% ethanol until analysis. Then, fixed tissues were embedded in paraffin by standard procedures and blocks were sectioned and stained with H&E. Serial sections of the lung (at least 10 sections spaced 100 μm) were stained with H&E, and metastatic foci were counted. Metastases were classified as small (<200 μm diameter), medium (between 200 and 400 μm diameter), and large (>400 μm diameter).

### Survival analysis

KM-plotter [22] (www.kmplot.com) was used to assess the effect of *USP54* expression on survival of intestinal cancer patients.

## References

[1] Lopez-Otin C, Bond JS (2008). Proteases: multifunctional enzymes in life and disease. J Biol Chem.

[2] Mason SD, Joyce JA (2011). Proteolytic networks in cancer. Trends Cell Biol.

[3] Quiros PM, Langer T, Lopez-Otin C (2015). New roles for mitochondrial proteases in health, ageing and disease. Nat Rev Mol Cell Biol.

[4] Turk B (2006). Targeting proteases: successes, failures and future prospects. Nat Rev Drug Discov.

[5] Drag M, Salvesen GS (2010). Emerging principles in protease-based drug discovery. Nat Rev Drug Discov.

[6] Freije JM, Fraile JM, Lopez-Otin C (2011). Protease addiction and synthetic lethality in cancer. Front Oncol.

[7] Lopez-Otin C, Matrisian LM (2007). Emerging roles of proteases in tumour suppression. Nat Rev Cancer.

[8] Clague MJ, Heride C, Urbe S (2015). The demographics of the ubiquitin system. Trends Cell Biol.

[9] Komander D, Clague MJ, Urbe S (2009). Breaking the chains: structure and function of the deubiquitinases. Nat Rev Mol Cell Biol.

[10] Fraile JM, Quesada V, Rodriguez D, Freije JM, Lopez-Otin C (2012). Deubiquitinases in cancer: new functions and therapeutic options. Oncogene.

[11] Quesada V, Diaz-Perales A, Gutierrez-Fernandez A, Garabaya C, Cal S, Lopez-Otin C (2004). Cloning and enzymatic analysis of 22 novel human ubiquitin-specific proteases. Biochem Biophys Res Commun.

[12] Stegmeier F, Sowa ME, Nalepa G, Gygi SP, Harper JW, Elledge SJ (2007). The tumor suppressor CYLD regulates entry into mitosis. Proc Natl Acad Sci U S A.

[13] Kon N, Kobayashi Y, Li M, Brooks CL, Ludwig T, Gu W (2010). Inactivation of HAUSP *in vivo* modulates p53 function. Oncogene.

[14] Xiao H, Wang LM, Luo Y, Lai X, Li C, Shi J, Tan Y, Fu S, Wang Y, Zhu N, He J, Zheng W, Yu X, Cai Z, Huang H (2016). Mutations in epigenetic regulators are involved in acute lymphoblastic leukemia relapse following allogeneic hematopoietic stem cell transplantation. Oncotarget.

[15] Merlos-Suarez A, Barriga FM, Jung P, Iglesias M, Cespedes MV, Rossell D, Sevillano M, Hernando-Momblona X, da Silva-Diz V, Munoz P, Clevers H, Sancho E, Mangues R, Batlle E (2011). The intestinal stem cell signature identifies colorectal cancer stem cells and predicts disease relapse. Cell Stem Cell.

[16] Sabates-Bellver J, Van der Flier LG, de Palo M, Cattaneo E, Maake C, Rehrauer H, Laczko E, Kurowski MA, Bujnicki JM, Menigatti M, Luz J, Ranalli TV, Gomes V (2007). Transcriptome profile of human colorectal adenomas. Mol Cancer Res.

[17] Ciurea ME, Georgescu AM, Purcaru SO, Artene SA, Emami GH, Boldeanu MV, Tache DE, Dricu A (2014). Cancer stem cells: biological functions and therapeutically targeting. Int J Mol Sci.

[18] Dick JE (2008). Stem cell concepts renew cancer research. Blood.

[19] Suresh B, Lee J, Kim KS, Ramakrishna S (2016). The importance of ubiquitination and deubiquitination in cellular reprogramming. Stem Cells Int.

[20] Buckley SM, Aranda-Orgilles B, Strikoudis A, Apostolou E, Loizou E, Moran-Crusio K, Farnsworth CL, Koller AA, Dasgupta R, Silva JC, Stadtfeld M, Hochedlinger K, Chen EI, Aifantis I (2012). Regulation of pluripotency and cellular reprogramming by the ubiquitin-proteasome system. Cell Stem Cell.

[21] Fraile JM, Ordonez GR, Quiros PM, Astudillo A, Galvan JA, Colomer D, Lopez-Otin C, Freije JM, Puente XS (2013). Identification of novel tumor suppressor proteases by degradome profiling of colorectal carcinomas. Oncotarget.

[22] Gyorffy B, Surowiak P, Budczies J, Lanczky A (2013). Online survival analysis software to assess the prognostic value of biomarkers using transcriptomic data in non-small-cell lung cancer. PLoS One.

